# Optimization of Breeding Tools in Quinoa (*Chenopodium quinoa*) and Identification of Suitable Breeding Material for NW Europe

**DOI:** 10.3390/plants14010003

**Published:** 2024-12-24

**Authors:** Tim Vleugels, Chris Van Waes, Ellen De Keyser, Gerda Cnops

**Affiliations:** 1 Plant Sciences Unit, ILVO (Flanders Research Institute for Agriculture, Fisheries and Food), Caritasstraat 39, 9090 Melle, Belgium; ellen.dekeyser@ilvo.vlaanderen.be (E.D.K.); gerda.cnops@ilvo.vlaanderen.be (G.C.); 2 Plant Sciences Unit, ILVO (Flanders Research Institute for Agriculture, Fisheries and Food), Burgemeester Van Gansberghelaan 109, 9820 Merelbeke, Belgium; chris.vanwaes@ilvo.vlaanderen.be

**Keywords:** *Chenopodium quinoa*, morphological traits, productivity traits, crude protein content, SSR markers, NIRS, paternity analysis

## Abstract

Quinoa (*Chenopodium quinoa*) cultivation has become increasingly popular in NW Europe but little is known about the performance of contract-free varieties in this region. In this study, we phenotyped 25 quinoa varieties on a single-plant basis in a field trial in Belgium. In addition, we optimized breeding tools such as NIRS (near-infrared reflectance spectroscopy) to estimate the seed crude protein content and a multiplex PCR set to identify true F_1_ progeny from pair crosses. We identified 14 varieties with sufficiently early maturity, 17 varieties with plant height below 150 cm, 21 large-seeded varieties, four varieties with a crude protein content exceeding 15%, and two low-saponin varieties. A variety of seed colors and plant morphological traits was observed. Seed yield was not correlated with maturity, plant height or saponin content, but was negatively correlated with seed crude protein content. NIRS could accurately predict seed crude protein content with a determination coefficient of 0.94. Our multiplex SSR set could correctly identify the paternity in 77% to 97% of progeny, depending on the pair cross. In conclusion, our study identified various contract-free varieties that may be suitable for cultivation in NW Europe. In addition, our study provides valuable phenotypic information and breeding tools that breeders can harness for breeding efforts in NW European quinoa.

## 1. Introduction

Quinoa (*Chenopodium quinoa*) originated in the Andes region of South America, where it has been cultivated for almost 7000 years [1]. This pseudocereal crop shows exceptional resilience to challenging environmental conditions such as frost, drought and salinity [2,3]. Quinoa exhibits substantial phenotypic variability, including the pigmentation and morphology of its inflorescences, panicles, leaves and seeds, as well as the diversity in maturity [1,3]. Quinoa seeds come in a variety of colors, including beige, orange, red, brown and black [1]. Quinoa is a highly nutritious food, with crude protein contents between 8% and 18% in a well-balanced composition that includes all essential amino acids in sufficiently high quantities [4,5]. In addition, quinoa is rich in minerals [6,7] and gluten-free, rendering it an excellent cereal alternative for people with celiac disease [8,9]. Most quinoa varieties contain saponins, a bitter-tasting compound, in the seed pericarp, which is disliked by consumers [1,4]. Quinoa varieties are classified as low, medium or high-seed saponin-containing based on foam tests [10,11]. Although it is possible to reduce saponin content through breeding [12], few saponin-free quinoa varieties have been developed so far [1,9].

In recent years, quinoa cultivation has gained popularity in NW Europe as a valuable crop for diversifying farmers’ crop rotations, enhancing soil health and boosting agrobiodiversity, as well as meeting the increasing demand for locally produced plant-based proteins [13,14]. NW Europe is in the northern maritime agro–climatic zone, which is characterized by moderately cool or cold winters and fairly mild summer temperatures with relatively wet winters and wet to occasionally dry summers [15]. The proximity to the coast results in a rather windy climate [15]. NW European soils are mostly sandy, loamy or clay types that are referred to as albeluvisol, cambisol, fluvisol and luvisol [16]. In NW Europe, agriculture is generally intensive. As a consequence, arable land is generally highly fertile due to traditionally high N fertilization but often suffers from soil compaction due to intensive soil management [16]. In some regions, soil acidification occurs as a result of atmospheric acid deposition and/or the use of synthetic fertilizers [16].

Cultivating quinoa in NW Europe presents challenges [9,13]. Early varieties that mature within a growing period of 150 days are considered most suitable as, later in autumn, low temperatures and short days slow maturation down and increased moisture complicates harvesting [13]. In addition, quinoa varieties should be medium-short, sparsely branched and exhibit good lodging tolerance to facilitate mechanical harvesting in the windy NW European climate [13]. Varieties should have good resistance to lodging, as lodging prevents panicles from drying after rainfall events and increases the risk of pre-harvest sprouting and molding while complicating the harvest [1]. Varieties with medium-dense panicles are most suitable for the moist NW Europe climate [13]. Dense, glomerulate panicles usually contain more seeds [3] but also require more time to dry after rainfall and morning dew, which renders them more sensitive to pre-harvest sprouting. Finally, mild summer temperatures and high humidity levels favor downy mildew disease caused by *Peronospora variabilis* and call for varieties with increased resistance to this disease [17].

Today, only a handful of quinoa varieties suitable for cultivation in NW Europe are commercially available, and there are no contract-free varieties that produce saponin-free seeds with colors different from beige or white [4,7]. Quinoa breeding efforts for NW Europe should focus on developing early, short varieties with medium-dense panicles that present a high and stable yield over growing seasons [9,13]. In addition, the European food industry prefers saponin-free varieties, as removing saponins adds additional processing costs prior to consumption [4,13]. While small-seeded varieties are suitable to make flour and processed products, European consumers prefer big quinoa seeds for their attractiveness in whole-seed food products such as salads. However, few large-seeded quinoa varieties are available for cultivation in NW Europe. Finally, quinoa is considered a protein crop in Europe [4], and policy makers and the food sector typically set a ‘protein crop’ threshold of 15% crude protein, rendering the seed crude protein content an important breeding trait [4,9].

Quinoa is predominantly self-pollinating and varieties are typically created through inbred lines [1]. The first step in breeding is the identification of promising material to initiate pair crosses [1,18]. While variety performances have been compared with the aim to identify promising breeding material in various regions around the world, e.g., in the USA, China, Colombia, Spain and Germany [3,7,8,19,20], variety performance at the plant level has not been studied in NW Europe. Subsequently, pair crosses are made between promising genotypes, and true F_1_ progeny are identified through phenotypic or genetic markers [8,9]. Microsatellite or simple sequence repeat (SSR) markers, for example, have been characterized in quinoa [1] and have been used successfully for paternity analysis in multiplex PCR reactions in other crops such as red clover [21] and sugarcane [22]. However, to our knowledge, no molecular paternity test is published for quinoa. Further generations of selection and self-pollination will eventually lead to candidate varieties [18]. Given the importance of seed crude protein content as a breeding trait, the small quantities of seeds available during breeding and the labor-intensive nature of lab protein analysis, nondestructive, high-throughput measuring techniques are to be preferred. The potential of NIRS (near-infrared reflectance spectroscopy) to estimate crude protein has been explored in previous quinoa studies [5], including studies on unmilled seeds [19,23]. Nonetheless, the potential of NIRS remains to be validated in quinoa material relevant for NW European conditions.

This study was performed in the context of breeding activities at ILVO, aiming to create new, contract-free varieties meeting the demands for cultivation in NW Europe. In this study, we screened a panel of 25 potentially promising quinoa varieties in a spaced-plant trial in Belgium. The objectives of our study were (1) to phenotype a relevant panel of 25 quinoa varieties for morphological, phenological and productivity traits in order to identify suitable material for cultivation and breeding efforts in NW Europe; (2) to optimize a NIRS calibration to estimate seed crude protein content on cleaned, unmilled quinoa seeds; and (3) to develop and validate a multiplex set of SSR markers to be used for paternity analysis in quinoa breeding.

## 2. Results

### 2.1. Weather Conditions During the Trial

**Figure 1:**
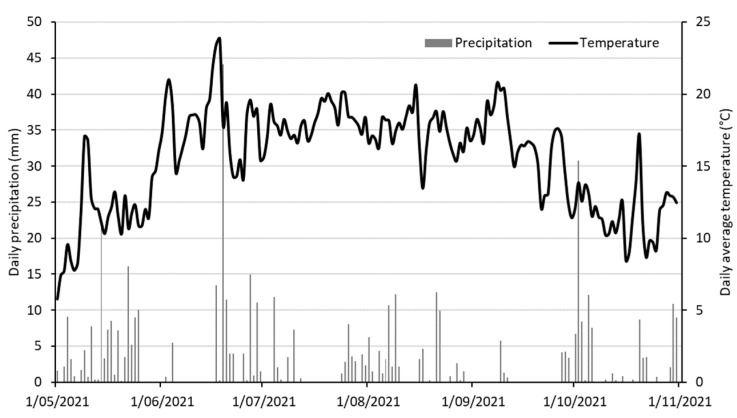
Daily average temperatures (black line, right axis) and daily precipitation (gray bars, left axis) for the trial site during the growing season of 2021, obtained from the Royal Meteorological Institute (KMI) in the form of interpolated data from nearby weather stations.

Daily average temperatures and daily precipitation for the trial site during the growing season of 2021 are presented in Figure 1. The year 2021 was generally a wet year in Belgium. Daily average temperatures in 2021 were 0.3 °C short of the long-term average of 11 °C [24]. The yearly total precipitation was 1039 mm—201 mm higher than the long-term average [24]. The year 2021 contained 20 days of sunny, warm weather, which is 10 days below the long-term average [24].

### 2.2. Screening of Quinoa Varieties in the Field

Phenotypic observations were obtained successfully for most plants, except for 20 plants that died during the trial; the seeds of these plants were not harvested and phenotypic observations were omitted. A total of 17 plants were lodged severely; these plants were tied to a bamboo stick to keep them upright and phenotyped for all traits except plant height at seed harvest. Except for a minor outbreak of aphids that was treated successfully, no pest infestations were observed. Downy mildew disease was observed with varying intensity depending on the variety, but was not considered further.

Most varieties displayed green foliage color, while only seven varieties contained plants with red or purple leaves. Varieties were generally homogeneous in foliage glaucosity; 11 varieties contained exclusively plants without hairs while 14 varieties displayed medium to strong glaucosity. Different stem colors were observed. ‘Peppermint’ showed a white stem color, eight varieties showed only green stems, nine varieties showed only purple stems, and the remaining varieties showed a mix of green and purple stems. Stem stripes and axil pigmentation were present in at least some plants of 13 and 10 varieties, respectively. Varieties were homogeneous for leaf size with six varieties displaying small leaf size, twelve displaying medium leaf size and seven displaying large leaf size, with varying degrees of leaf dentation. The frequencies of scores per variety for leaf and stem morphological traits are presented in Table 1.

**Figure 2:**
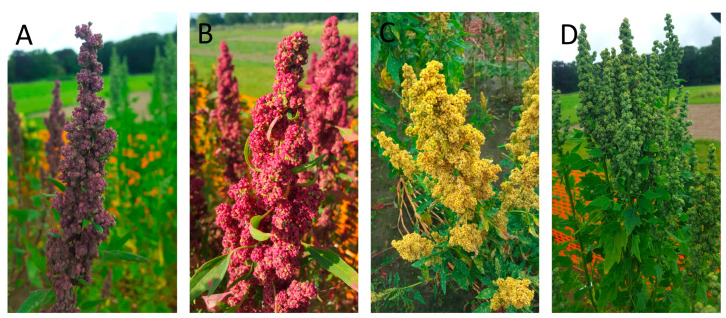
Example of the phenotypic diversity observed in panicle traits among our panel of varieties and plants: narrow (**A**), intermediate (**B**,**C**) to broad (**D**) panicle width; dense (**A**), intermediate (**B**,**C**) to loose (**D**) panicle density; and purple (**A**), red (**B**), yellow (**C**) or green (**D**) panicle color.

The screened material was homogeneous for growth habit. Only five varieties displayed no or sparse branching, the most preferred growth habit by breeders, while twenty varieties displayed substantial branching. The varieties had a wide range of inflorescence colors, including white, green, pink and purple, as well as panicle dimensions (Figure 2). Nonetheless, the varieties were generally homogeneous for panicle dimensions; all accessions had glomerulate panicles with medium (14) to broad (11) width and loose (9), medium (11) or dense (3) panicle density. Various panicle colors were observed (white, purple, red, pink, yellow, orange, brown and green), and the varieties often presented plants with different colors. A total of 598 single-plant seed lots were harvested between August 3 and October 11; i.e., 116 to 174 days after sowing. A color inspection of the harvested seeds revealed six varieties with exclusively white-seeded plants and twelve with predominantly beige-seeded plants. Five varieties contained red-seeded plants, often mixed with brown- or beige-seeded plants. Only ‘Black Quinoa’ and ‘Colorado Black Shelly 25’ contained plants with black seeds. ‘Cocoa Cherry’, ‘Pink Nugget’ and ‘Red Nugget’ plants produced also pink pigments in their beige or brown seeds. The frequencies of scores for growth habits and panicle morphological traits are presented in Table 2.

In addition, phenological and productivity traits were investigated. Our panel of varieties headed 63 to 90 days after sowing (DAS) and matured 116 to 174 days DAS. Plant heights at seed harvest were between 90 and 184 cm. Varieties yielded between 39 g and 137 g seeds per plant, with average TSW (thousand-seed weight) between 1.6 g and 3.6 g, and average saponin contents producing between 0.8 cm and 5.7 cm of foam [10]. Two varieties, ‘Mint Vanilla’ and ‘Magenta Sunset’, were identified with low saponin (<1 cm), ‘White Nugget’ was a high-saponin variety and the remaining varieties had average seed saponin content according to Ward [11]. Nonetheless, all varieties showed at least one individual plant with low seed saponin content, with a total of 162 plants in the trial. Varieties displayed average seed crude protein contents between 12.1% and 16.1%, with an average of 13.9%. Table 3 presents the variety averages for phenological and productivity traits.

Pearson correlation coefficients between traits are given in Table 4. Heading date and maturity date were moderately correlated (r = 0.49, *p* < 0.05). Plant height at seed harvest was moderately correlated with the heading date (r = 0.73, *p* < 0.05) and maturity date (r = 0.63, *p* < 0.001). Seed yield per plant was strongly and negatively correlated with crude protein content (r = −0.60, *p* < 0.001). Crude protein content and saponin content were positively correlated (r = 0.43, *p* < 0.05). Correlations between heading date, maturity date, plant height saponin content and TSW and seed yield per plant were low and not significant.

### 2.3. Paternity Analysis Through Multiplex SSR Set

To optimize molecular paternity analysis, 12 SSR markers with good amplification and sufficient polymorphisms in a preliminary test were evaluated in three experimental multiplex PCR sets a, b and c, with each set containing four markers (Table 5). In experimental sets a and c, amplification was satisfactory, and no difference was noted between the amplified fragments in the single PCR reactions vs. the multiplex reactions. Set b was not satisfactory; amplification was poor and amplified fragments from markers QATG019, QCA037 and KGA003 had shifted 1 to 2 bps compared to the single PCR reactions, indicating possible interaction between markers. The final multiplex SSR set was made by combining five polymorphic and compatible markers from experimental sets a and c: KGA027^NED^, QAAT050^VIC^, QAAT076^PET^, QAAT024^FAM^ and QCA071^FAM^ (fluorescent labels in superscript); the latter two markers had the same label but different amplification range.

The final multiplex SSR set was validated on 844 progeny plants from 91 pair crosses between five pairs of varieties, which were performed as part of breeding work (not further described) that emanated from this study. Depending on the variety pair, our multiplex set correctly identified the paternity between 92% and 97% of progeny plants in variety pairs 1, 3, 4 and 5 (Table 6). In variety pair 2, three markers generally showed low amplification, resulting in a substantially lower success rate of 77%. Depending on the variety pair, between 9% and 41% of progeny plants resulted from cross-pollination. These true F_1_ plants were used in further breeding activities, which are outside of the scope of this paper.

## 3. Discussion

### 3.1. Phenotypic Screening of Varieties

The material screened herein displayed extensive variation in morphological traits, which is illustrative for a diverse species such as quinoa [3,8]. Five varieties, ‘Biobio’, ‘Buffy’, ‘Cherry Vanilla’, ‘Cocoa Cherry’ and ‘Fingerhead GP’, displayed no or sparse branching, while the remaining material was substantially branched. Varieties were homogeneous for panicle shape and dimensions. All accessions produced glomerulate panicles that were medium (14) to broad (11) in size. Panicles with loose (9) or intermediate density (13) were most common, but ‘Incred White’, ‘Kaslala’ and ‘Temuco’ produced denser panicles. A plethora of inflorescence colors, foliage colors and panicle colors were observed, with up to five panicle colors noted in some varieties. In addition, multiple seed colors were observed, with white or beige seeds the prevailing color. Red and brown seeds were less commonly observed, and only two accessions, ‘Black quinoa’ and ‘Colorado Black Shelly 25’, exhibited black seeds. Varieties with red or black seeds often also contained plants producing beige seeds, indicating that they were not genetically fixed for seed color. Quinoa breeders can harness the morphological and productivity trait data for this panel of varieties to identify suitable starting material for breeding purposes.

Most varieties performed well under NW European conditions. Heading date was between 63 and 90 DAS, which is similar to what is described by Emrani et al. [8] in N Germany. The maturity date was between 116 and 174 DAS, congruent to previous studies in Belgium and SW Germany [4,7], where the climate is not so different. In NW Europe, varieties should ideally mature no later than 150 DAS to allow for harvesting between mid-August and mid-September [4,13]. A total of 14 accessions matured within this timeframe. The earliest variety ‘Zeno’ matured 116 DAS, slightly earlier than in SW Germany (122 to 125 DAS) [7], but also presented distinctly low seed yield compared to the remaining material in our panel. ‘Zeno’ appears to be well adapted to short growing seasons but may not achieve its full yield potential in Belgium.

Resistance to lodging is considered important to ensure good seed filling and minimal harvest loss and is influenced by stem strength and plant height [1,20]. Our panel of quinoa varieties presented substantial differences in plant height, between 90 and 182 cm. This is lower than plant heights reported by previous studies in N Germany (113–218 cm) [8], China (137–280 cm) [18] and Colombia (177 cm on average) [3], but this may be due to the preselection of material for this study—tall accessions were deliberately not included in this study. Two varieties, ‘Fingerhead GP’ and ‘Pink Nugget’, reached higher than 170 cm, rendering them less desirable for NW European conditions [13]. ‘Zeno’ was the shortest variety with 90 cm at seed harvest. While none of the screened accessions was entirely saponin-free, we observed two varieties, ‘Magenta Sunset’ and ‘Mint Vanilla’, with low saponin content (<1 cm foam).

Intra-variety variation for saponin content was considerable. We observed no fewer than 162 low-saponin plants spread over our panel. In 13 varieties, at least five individual plants with low saponin content were found, which opens perspectives for quinoa breeders using this material for low-saponin breeding efforts [12]. The TSW was generally high in our study, between 1.6 g and 3.6 g, similar to that observed by De Bock et al. [4] and Emrani et al. [8] (1.9 to 3.7 g and 1.5 to 4.9 g, respectively). Only two varieties displayed a TSW lower than 2 g, which sharply contrasts the material screened in China where more than 90% of varieties presented a TSW lower than 2 g [20]. Six varieties, ‘Black Quinoa’, ‘Colorado Black Shelly 25’, ‘French Vanilla’, ‘Oro de Valle’, ‘Peppermint’, ‘Red Head’ and ‘Zeno’ exceeded 3 g TSW, which agrees with the SW German study that also noted high TSW for ‘Zeno’ (3.3 g) [7]. These varieties are interesting for meeting consumer preferences in NW Europe and may be valuable starting material for breeding efforts aiming at large-seeded plants.

Our trial displayed an average seed crude protein content of 13.9%, with a range between 12.1% and 16.1%, and four varieties attained the ‘protein crop’ threshold of 15% crude protein. This protein range is similar to other recent studies in European regions with similar climates [4,6,7] but smaller compared to previous studies in the USA and Colombia [19,27]. Possibly, seed protein contents in our study were relatively low due to the modest N fertilization (53 kg/ha) provided [28]. The recommended N fertilization for quinoa cultivation in NW Europe is between 90 and 130 kg/ha N [4,8]. Although our trial was a spaced-plant trial, which typically means that less fertilization is provided than in a mini-plot trial, our N fertilization may have been lower than optimal. Our study confirmed that NIRS is a suitable method to estimate quickly and accurately the crude protein content of intact, unmilled quinoa seeds. The coefficient of determination (R^2^) of our NIRS calibration for seed crude protein was 0.94, which is substantially higher than the value reported by Ferreira et al. [23] and only slightly lower than the calibration by Craine et al. [19], who screened an extensive collection of samples (0.77 and 0.97 for both studies, respectively). Hence, the NIRS calibration presented herein was a reliable technique to estimate seed crude protein content in this present panel of varieties, which opens perspectives for quinoa breeders to use NIRS for crude protein content in their selection programs.

Correlation analysis revealed that varieties with later flowering and maturation generally grew taller. This was also found by previous studies in the USA and N Germany [8,19] and suggests that varieties with later maturation invest more in vegetative growth before initiating flowering and often continue vegetative growth during the flowering and seed maturation stage, allowing them to grow taller [1]. In contrast to previous studies, this present study did not find a significant relation between seed yield and heading date, which is congruent with the different relations observed in previous studies between these traits, ranging from negative [19], positive [3,8], to no correlation [20]. In contrast to what was expected, our results did not suggest that early-heading varieties yielded more seeds in our field trial in Belgium. Possibly, this may be explained by the preselection of material for this study, as late accessions were deliberately not included. Plants that yielded more seeds generally also tended to produce bigger seeds in our study, which is congruent with previous studies that all found a strong correlation between seed yield and TSW [3,8,19,20]. Finally, our study confirmed the negative correlation between seed yield and seed crude protein content [4,6,19]. This negative relation between yield and protein content is expected, as higher protein levels usually result from lower carbohydrate accumulation in the kernel, linking increased seed protein contents with lower yield [1]. However, genetic factors also influence seed protein content [1], and while the yield-protein trade-off exists, it is variety-dependent [4]. This suggests that seed protein contents can be improved through breeding [1]. However, quinoa breeders should be cautious, as focusing solely on yield could inadvertently reduce seed protein content [19]. Instead, they should aim to select plants that combine high seed yield with adequate protein content. Finally, we found no correlation between seed yield and seed saponin content, which indicates that breeders can increase yield and reduce saponin content simultaneously.

The results obtained for the varieties screened in this study should be interpreted carefully, as our field trial did not have randomized repetitions. In addition, the results were obtained in one growing season only, which may have biased variety performance. The growing season of 2021 was generally wet, with slightly lower average temperatures, considerably more precipitation and fewer warm, sunny days than the long term average [24]. Drought stress did not manifest itself in our field trial. As a consequence, varieties that develop well under colder, wetter conditions could have been favored. Drought-tolerant varieties, on the other hand, had no advantage in our trial and their performance may have been underestimated. The cool and wet conditions in our trial favored the development of downy mildew (*Peronospora variabilis*), which is known to interact with seed yield [9,17]. Varieties with increased resistance likely benefited, while varieties with poor disease resistance may have suffered from yield reduction as a consequence of the disease pressure in our trial. As a consequence, our results should be considered informative but preliminary.

### 3.2. Molecular Paternity Analysis Through SSR Markers

A key step in quinoa breeding is making pair crosses between varieties or genotypes with desirable and complementary traits [9]. The difficult aspect of this step is discriminating between true F_1_ progeny from cross-pollinations and progeny from self-pollinations [29]. Preferably, pair crosses are designed so that simply inherited traits such as foliage color, leaf axil pigmentation or seed color can be used as phenotypic markers to pinpoint true F_1_ progeny [29]. In this sense, the morphological characterization of varieties described herein provides valuable information for breeders aiming to use varieties from our panel in their crosses. When it is not possible to use phenotypic markers to identify the F_1_ progeny, molecular markers must be used instead. Molecular markers are the subject of numerous publications in the context of genetic diversity, marker-assisted selection or, more recently, genomic selection [30]. While some publications describe molecular paternity analysis in cross-pollinating crops [21,22], publications rarely report on paternity analysis in self-pollinating crops, despite its common use in breeding.

In this study, we designed a multiplex SSR set for molecular paternity analysis. From the twelve markers that were tested in our experimental multiplex sets, roughly half (7) showed different alleles in the three varieties studied (Table 5). A similar trend was observed in the validation of our multiplex SSR set. In the five series of crosses, two to three out of five markers were discriminative. For paternity analysis, however, it suffices that parents display different alleles at one locus. Our multiplex SSR set was highly successful; in four out of five series of crosses, the paternity could be correctly assigned for at least 92% of progeny plants. A considerable proportion of progeny with correctly assigned paternity resulted from cross-pollinations and were used in breeding efforts emanating from this work, between 9% and 41%, depending on the variety pair.

Our results indicate that multiplex SSR sets can efficiently and reliably assess the paternity of F_1_ progeny plants, with potential for broader application beyond the material screened in this study. Previous studies in cross-pollinating crops report similar success rates for paternity analysis but they often used more markers. In diploid red clover (*Trifolium pratense*), multiplex sets with 18 SSR markers resulted in a success rate of 97% [21]. In sugarcane (*Saccharum officinarum*), seven SSR markers could identify the paternity in 79 to 99% of the progeny from a seven-parent polycross [22]. Finally, our screening of the 12 markers in experimental multiplex sets also hinted at interesting information on the degree of homogeneity of the varieties tested (Table 5). ‘Zeno’ plants displayed one unique allele at ten loci, compared to nine loci in ‘Oro de Valle’ and only two loci in ‘Black Quinoa’. This suggests a higher degree of homogeneity or genetic fixation in the former two varieties compared to ‘Black Quinoa’, which appears to be more genetically heterogeneous.

In conclusion, the characterization of our panel of varieties allows farmers and advisors to pinpoint suitable, contract-free varieties for cultivation in NW Europe. Although our results should be regarded as preliminary, they allow breeders to identify suitable material for breeding efforts towards improved performance in NW European conditions. Varieties with optimal maturity date and seed yield did not necessarily present optimal quality traits such as TSW, saponin content and protein content, which demonstrates the importance of breeding for adapted cultivars. The NIRS method for crude protein content and the multiplex SSR set for paternity analysis should pave the way for future breeding efforts. Interesting varieties from our variety panel have already been used for crosses in our breeding program, and candidate varieties with increased adaptation should be available within a few years.

## 4. Materials and Methods

### 4.1. Plant Material

A panel of 25 potentially promising quinoa varieties was composed of sufficient early-maturation and acceptably low plant height based on the information of the supplier. Details of the plant material are presented in Table 7. Our panel included material originating mainly from the USA but also from Bolivia, Chile and Europe. The farm-original varieties ‘Black Quinoa’, ‘Kcoito’ and ‘Oro de Valle’ were selected for the Belgian climate by De Nieuwe Tuin. The trial was performed in the growing season of 2021. Seeds of the 25 varieties were sown in early spring in Quickpot^®^ trays (HerkuPlast QP96T) on 9 April, resulting in 48 seedlings per variety. Seedlings were grown in a naturally heated greenhouse and planted in a sandy loam field on 11 May at the ILVO facilities in Melle, Belgium (50.99° N, 3.78° E, 23 m asl). Temperature and precipitation data for the trial site were available from the Royal Meteorological Institute (KMI) in the form of interpolated data from nearby weather stations, with the nearest station about 4 km away at 50° 58′49″ N, 3°48′57″ E.

### 4.2. Field Trial Setup

The trial was set up as a spaced-plant trial with each variety arranged in a row of 25 spaced plants. Planting distance was 1 m between rows and 0.4 m between plants within rows. Border rows sown with the variety ‘Zeno’ were included on both sides of the trial to minimize border effects; no measurements were made on these rows. The total dimensions of the trial were 30 m by 10 m. After planting, a synthetic fertilizer was applied, providing a modest fertilization of 56/16/120 NPK units. Weeds were controlled manually by weeding and hoeing on a regular basis. A single insecticide application was performed on 18 June (deltamethrin, 10.0 g ha^−1^ a.i.) (Split; Bayer Crop Science, Diegem, Belgium) to control a minor infestation of aphids. Throughout the growing season, plants were phenotyped for morphological traits (foliage color, foliage glaucosity, stem color, presence of stem stripes, pigmentation of leaf axil, leaf size, leaf dentation, growth habit, inflorescence color, panicle shape, panicle width, panicle density and panicle color) following the methodology described by Stanschewski et al. [25]. In addition, phenological data were recorded; plants were considered to have headed when panicles were larger than 1 cm and considered mature when the newly formed seeds were ready to be harvested [25]. Individual plants were harvested for seeds at their maturity date, and seed lots were cleaned using a small threshing machine designed for small seed lots. The seed weight of each single-plant seed lot was determined, and the seed color was determined by visual inspection [25]. The TSW was calculated from the average of weighing 100 seeds four times. Seed saponin content was estimated by measuring the foam formation in cm on a sample of 0.5 g seeds [10]. Seed protein content was estimated through NIRS, which is explained further. A detailed overview of the traits, scales and observation dates observed in this study is given in Table 8. To detect possible differences between varieties for phenological and productivity traits (heading date, maturity date, plant height at harvest, seed yield per plant, TSW, seed saponin content and seed protein content), one-way ANOVA was performed using the plants within each variety as repetitions. Least significant difference (LSD_d_) was calculated according to Al-Fahham [26]. In addition, Pearson correlation coefficients were calculated for each productivity trait. Statistics were carried out in R statistical software version 4.2.2 [31], implemented in RStudio [32]. Correlations were considered significant at *p* < 0.05.

### 4.3. NIRS for Crude Protein Content

**Figure 3:**
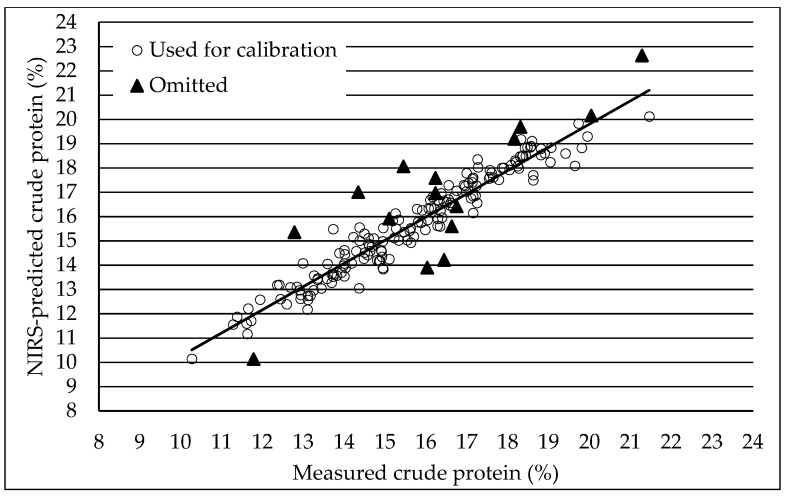
Measured crude protein content and NIRS-estimated crude protein content for 178 quinoa seed samples used in the calibration, with indication of the 163 samples that were used in the final calibration (circles) and the 18 outlier samples (triangles) that were omitted.

Analyses of crude protein content were performed using NIRS on cleaned, unmilled seeds. The samples were scanned with a FOSS XDS monochromator instrument with ISIscan v2.85.1 software. The inverse reflectance (log (1/R)) was collected from 400 to 2500 nm in steps of 2 nm. The NIRS calibration was based on 109 seed samples from previous quinoa trials in Belgium and 69 samples from the current trial. Principal component analysis was used to select samples to achieve maximal coverage of the spectral variation of the sample sets [33]. Like this, 178 samples from 4 years (2018–2021) were selected for the chemical reference analyses of crude protein (ISO 5983-2). The calibration development was executed with WINSI v4.9.0. software using modified partial least squares regression [34]. Cross-validation was used to select the optimum number of partial least squares. After outlier elimination, 163 samples remained in the calibration (Figure 3). The standard error of calibration and cross-validation was 5.5 and 6.6 g/kg for crude protein, respectively. The determination coefficient (R^2^) of the linear regression between reference values and NIRS-predicted values of the calibration set was 0.94. Using the developed calibration, the crude protein content was predicted for 588 samples in the current study.

### 4.4. Molecular Work

To optimize molecular paternity analysis, we considered 18 SSR markers with a high degree of polymorphism and known compatibility with a balanced set of fluorescent labels (FAM, VIC, NED and HEX) identified by previous studies [35,36,37,38,39] (Table 9). In a preliminary analysis, these markers were screened on eight varieties: ‘Black quinoa’, ‘Chadmo’, ‘Kaslala’, ‘Incred White’, ‘Oro de Valle’, ‘Pink Nugget’, ‘Red Head’ and ‘Zeno’. From each variety, five single-plant DNA samples and two pooled samples containing the DNA of five plantlets were tested, along with a negative control (sterile water). DNA was extracted from 20 mg leaf material of young plantlets using the CTAB protocol [40]. The amplification of each marker was inspected visually, and 12 markers that showed good amplification and sufficient polymorphisms were selected for testing in multiplex PCR reactions. Three experimental multiplex PCR sets, each containing four markers with different fluorescent labels (Table 9), were tested on three varieties ‘Black Quinoa’, ‘Oro de Valle’ and ‘Zeno’, with five single-plant DNA samples per variety. In parallel, the 12 markers were tested in separate PCR reactions. Primers were coupled with the fluorescent labels used in the literature [35], except for HEX labels, which were replaced by PET labels in our study (Table 9). Single PCR reactions were carried out using a generic forward primer (FAM labeled) and a 5′ tail-DNA (ACGACGTTGTAAAA) added to all F primers as described in Hayden et al. [41]. The reaction mixture consisted of 30 ng DNA in a total volume of 25 µL using 1U GoTaq^®^ G2 Flexi DNA polymerase, 1X colorless GoTaq^®^ Flexi buffer, 1.5 mM MgCl_2_ (all Promega Benelux B.V., Leiden, the Netherlands), 0.2 mM dNTP, 0.5 µM generic forward primer (5′ FAM- ACGACGTTGTAAAA), 0.05 µM SSR specific forward and 0.5 µM reverse primer (Table 9). PCR was performed in a GeneAmp™ 9700 PCR system (Applied Biosystems; Fisher Scientific S.R.L., Merelbeke, Belgium) using the following temperature profile: 95 °C 10 min, 15 cycles of 95 °C 45 s, 72 °C with a touchdown to 58 °C at a ramp rate of 1 °C/cycle, 72 °C 30 s, followed by 35 cycles of 95 °C 45 s, 58 °C 45s and 72 °C 30 s. Multiplex PCR reactions contained 5 µL QIAGEN 2X Multiplex PCR Master Mix (Qiagen Benelux B.V., Venlo, the Netherlands), 0.2 µL forward primer and 0.2 µL reverse primer for each marker in the multiplex set, as well as 1 µL DNA in a total volume of 10 µL. Multiplex PCR reactions were performed in a GeneAmp™ 9700 PCR system (Applied Biosystems; Fisher Scientific S.R.L., Merelbeke, Belgium) as follows: 95 °C 15 min, followed by 27 cycles of 94 °C 30 s, 57 °C 90 s and 72 °C 60 s, and a final extension of 30 min at 60 °C. SSR fragments were analyzed with capillary electrophoresis on an ABI Prism 3730*xl* genetic analyzer (Applied Biosystems; Fisher Scientific S.R.L., Merelbeke, Belgium) using the genRES^®^ Ls500-ORN size standard. GeneScan^®^ v.3.1.2 analysis software (Applied Biosystems; Fisher Scientific S.R.L., Merelbeke, Belgium) was used to translate the information into fragment-sizing information, and alleles were scored with GeneMapper^™^ v6.0 (Applied Biosystems; Fisher Scientific S.R.L., Merelbeke, Belgium).

The three experimental multiplex sets were evaluated for compatibility and interaction between markers, and five polymorphic markers were combined into the final multiplex SSR set. This multiplex SSR set was validated in breeding work that emanated from this study. From our field trial, we selected 31 promising single-plant seed lots from varieties ‘Biobio’, ‘Black Quinoa’, ‘Cherry Vanilla’, ‘French Vanilla’, ‘Mint Vanilla’, ‘Peppermint’ and ‘Zeno’. From these seed lots, parent plants were grown to be used in five series of pair crosses. Pair crosses were performed using a slightly modified method as described by Peterson et al. [29]. We performed 91 pair crosses between five varieties pairs, rendering 844 progeny plants that were screened with our multiplex SSR set to identify the paternity. DNA from parent plants was extracted using the CTAB method [40], while DNA from progeny plants was sampled using the FTA^®^ protocol (Whatmann; Carl Roth GmbH, Karlsruhe, Germany), a high-throughput method for DNA collection [42]. For this, young plantlets (2 to 3 weeks old) were sampled in the morning by crushing out cell sap from individual leaves with a pestle on 0.18 mm-thick Whatman chromatography paper; cards were air-dried and cleaned up with FTA^®^ Purification Reagent (Whatman; Carl Roth GmbH, Karlsruhe, Germany) according to the manufacturer’s protocol. For progeny PCR reactions, a 3 mm FTA card disk containing the DNA was directly added to the 10 µL PCR mixture and PCR was performed as described above. Paternity analysis was performed using the R-package ‘PolyPatEx’ [43] in R version 4.2.2. [31], implemented in RStudio [32]. Ploidy was set as a tetraploid, self-compatibility as true, the number of allowed mismatches as zero and the minimum number of ‘valid’ loci (loci for which data are available) as two. For each cross, the proportion of progeny plants for which the paternity could be correctly assigned was calculated. Progeny plants resulting from cross-pollinations (true F_1_ plants) were used in further breeding activities, and are not further described.

## Figures and Tables

**Table 1:** Frequencies of scores (% of plants) observed in each variety for leaf and stem morphological traits: foliage color, foliage glaucosity, stem color, presence of stem stripes, pigmentation of leaf axil, leaf size and leaf dentation, along with the number of plants screened per variety.

Variety	Foliage Color *	Foliage Glaucosity *	Stem Color *	Presence of Stem Stripes *	Pigmentation of Leaf Axil *	Leaf Size *	Leaf Dentation *	No. Plants Screened
Biobio	1 (100%)	1 (100%)	4 (100%)	1 (100%)	1 (100%)	5 (100%)	5 (100%)	25
Black Quinoa	1 (24%), 2 (56%), 5 (20%)	1 (100%)	2 (88%), 4 (12%)	1 (84%), 9 (16%)	1 (84%), 7 (16%)	3 (100%)	1 (100%)	25
Buffy	1 (100%)	1 (100%)	4 (100%)	1 (100%)	1 (100%)	5 (100%)	5 (100%)	25
Chadmo	2 (100%)	3 (100%)	2 (96%), 4 (4%)	1 (100%)	1 (96%), 7 (4%)	5 (100%)	1 (17%), 3 (42%), 5 (42%)	24
Cherry Vanilla	1 (100%)	1 (100%)	4 (100%)	1 (100%)	1 (100%)	5 (100%)	3 (100%)	24
Cocoa Cherry	1 (64%), 4 (4%), 5 (32%)	1 (100%)	4 (100%)	1 (100%)	1 (100%)	7 (100%)	3 (4%), 5 (96%)	25
Colorado Black Shelly 25	1 (8%), 2 (68%), 4 (4%), 5 (20%)	3 (100%)	2 (84%), 4 (16%)	1 (16%), 9 (84%)	1 (88%), 7 (12%)	3 (100%)	1 (28%), 3 (60%), 5 (12%)	25
Fingerhead GP	1 (61%), 5 (39%)	3 (91%), 5 (9%)	2 (65%), 4 (35%)	1 (70%), 9 (30%)	1 (61%), 7 (39%)	7 (100%)	1 (4%), 3 (35%), 5 (61%)	22
French Vanilla	1 (100%)	3 (100%)	2 (96%), 4 (4%)	1 (96%), 9 (4%)	3 (96%), 7 (4%)	7 (100%)	5 (100%)	25
Golden afternoon	1 (100%)	3 (100%)	2 (92%), 4 (8%)	1 (92%), 9 (8%)	1 (88%), 7 (12%)	7 (100%)	5 (100%)	25
Incred White	2 (100%)	3 (100%)	2 (100%)	9 (100%)	1 (100%)	5 (100%)	1 (12%), 3 (72%), 5 (16%)	25
Ivory	3 (100%)	1 (100%)	2 (100%)	9 (100%)	3 (100%)	7 (100%)	3 (23%), 5 (77%)	22
Kaslala	2 (67%), 3 (33%)	1 (100%)	2 (71%), 4 (29%)	9 (100%)	1 (71%), 7 (29%)	3 (100%)	1 (96%), 3 (4%)	24
Kcoito	1 (4%), 2 (79%), 4 (4%), 5 (13%)	1 (100%)	2 (100%)	9 (100%)	1 (100%)	3 (100%)	1 (100%)	24
Magenta sunset	2 (100%)	3 (100%)	2 (100%)	9 (100%)	1 (100%)	5 (100%)	1 (9%), 5 (91%)	22
Mint Vanilla	1 (100%)	3 (100%)	2 (100%)	1 (100%)	1 (100%)	7 (100%)	5 (100%)	25
Oro de Valle	1 (4%), 2 (96%)	1 (100%)	2 (100%)	9 (100%)	3 (100%)	5 (100%)	1 (100%)	24
Peppermint	1 (100%)	3 (100%)	1 (100%)	1 (100%)	1 (100%)	7 (100%)	5 (100%)	23
Pink Nugget	1 (20%), 4 (12%), 5 (68%)	3 (100%)	4 (100%)	1 (100%)	3 (100%)	3 (100%)	3 (100%)	25
Red Head	1 (100%)	3 (100%)	4 (100%)	1 (100%)	1 (100%)	5 (100%)	5 (100%)	23
Red Nugget	1 (14%), 4 (14%), 5 (73%)	3 (100%)	4 (100%)	1 (100%)	1 (100%)	5 (100%)	3 (100%)	22
Temuco	1 (4%), 2 (96%)	3 (100%)	2 (100%)	9 (100%)	1 (100%)	5 (100%)	5 (100%)	24
White Nugget	1 (100%)	3 (100%)	4 (100%)	1 (100%)	1 (100%)	5 (100%)	1 (8%), 3 (92%)	24
White Spike	1 (100%)	1 (100%)	4 (100%)	1 (100%)	1 (100%)	5 (100%)	3 (4%), 5 (96%)	25
Zeno	2 (100%)	1 (100%)	2 (100%)	1 (100%)	1 (100%)	3 (100%)	1 (100%)	24

* Traits were scores as described in Stanschewski et al. [25]. The actual meaning of the different scores for each trait is described in Table 8.

**Table 2:** Frequencies of scores observed per variety (% of plants) for growth habit and panicle traits: inflorescence color, panicle shape, panicle width, panicle density, panicle color and seed color, along with the number of plants screened per variety.

Variety	Growth Habit *	Inflorescence Color *	Panicle Shape *	Panicle Width *	Panicle Density *	Panicle Color *	Seed Color **	No. Plants Screened
Biobio	1 (100%)	2 (100%)	1 (100%)	5 (100%)	1 (100%)	3 (100%)	white	25
Black Quinoa	5 (100%)	2 (84%), 5 (8%), 6 (8%)	1 (100%)	7 (100%)	1 (100%)	2 (4%), 3 (8%), 4 (8%), 5 (56%), 10 (24%)	black, red	25
Buffy	1 (100%)	1 (100%)	1 (100%)	5 (100%)	1 (100%)	1 (4%), 3 (96%)	beige	25
Chadmo	5 (100%)	1 (96%), 6 (4%)	1 (100%)	7 (100%)	3 (100%)	5 (100%)	beige, red	24
Cherry Vanilla	1 (100%)	1 (96%), 5 (4%)	1 (100%)	5 (100%)	1 (100%)	1 (4%), 3 (92%), 5 (4%)	white	24
Cocoa Cherry	1 (100%)	2 (88%), 6 (12%)	1 (100%)	5 (100%)	1 (100%)	2 (12%), 3 (84%), 5 (4%)	beige-pink	25
Colorado Black Shelly 25	5 (100%)	2 (88%), 6 (12%)	1 (100%)	7 (100%)	3 (100%)	2 (20%), 3 (16%), 5 (20%), 10 (44%)	red, black, brown	25
Fingerhead GP	3 (100%)	1 (52%), 6 (48%)	1 (100%)	3 (4%), 5 (96%)	1 (100%)	2 (48%), 5 (43%), 10 (9%)	beige, brown	22
French Vanilla	5 (100%)	1 (100%)	1 (100%)	5 (100%)	1 (100%)	5 (100%)	beige	25
Golden Afternoon	5 (100%)	1 (100%)	1 (100%)	5 (100%)	3 (100%)	3 (64%), 5 (28%), 8 (8%)	beige	25
Incred White	5 (100%)	2 (100%)	1 (100%)	7 (100%)	5 (100%)	7 (100%)	white	25
Ivory	5 (100%)	2 (100%)	1 (100%)	7 (100%)	1 (100%)	5 (100%)	beige	22
Kaslala	5 (100%)	2 (71%), 6 (29%)	1 (100%)	7 (100%)	5 (100%)	2 (25%), 4 (46%), 5 (21%), 7 (4%), 10 (4%)	red, beige	24
Kcoito	5 (100%)	2 (96%), 6 (4%)	1 (100%)	7 (100%)	3 (100%)	4 (63%), 6 (38%)	red, beige	24
Magenta Sunset	5 (100%)	1 (91%), 5 (9%)	1 (100%)	7 (100%)	3 (100%)	1 (4%), 3 (96%)	beige	22
Mint Vanilla	5 (100%)	1 (100%)	1 (100%)	5 (100%)	3 (100%)	5 (100%)	white	25
Oro de Valle	5 (100%)	2 (100%)	1 (100%)	7 (100%)	3 (100%)	5 (46%), 6 (54%)	beige	24
Peppermint	5 (100%)	1 (100%)	1 (100%)	5 (100%)	3 (100%)	5 (100%)	white	23
Pink Nugget	5 (100%)	2 (96%), 6 (4%)	1 (100%)	5 (100%)	3 (100%)	2 (4%), 3 (96%)	brown-pink	25
Red Head	5 (100%)	2 (100%)	1 (100%)	5 (100%)	3 (100%)	5 (83%), 10 (17%)	beige	23
Red Nugget	5 (100%)	2 (100%)	1 (100%)	5 (100%)	3 (100%)	3 (100%)	brown-pink	22
Temuco	5 (100%)	2 (100%)	1 (100%)	7 (100%)	5 (100%)	1 (79%), 7 (21%)	beige	24
White Nugget	5 (100%)	2 (100%)	1 (100%)	5 (100%)	3 (100%)	3 (13%), 5 (88%)	beige, beige-pink	24
White Spike	5 (100%)	1 (100%)	1 (100%)	5 (100%)	3 (100%)	5 (100%)	beige	25
Zeno	5 (100%)	1 (100%)	1 (100%)	7 (100%)	1 (100%)	5 (100%)	white	24

* Traits were scores as described in Stanschewski et al. [25]. The actual meaning of the different scores for each trait is described in Table 8. ** When multiple seed colors occurred in a variety, the most prevalent color is listed first.

**Table 3:** Variety averages ± SD for phenological and productivity traits: heading date, maturity date, plant height at seed harvest, seed yield per plant, thousand-seed weight (TSW), seed saponin content, number of low-saponin plants (<1 cm in foam tests [10]) and seed crude protein content, along with the number of plants screened per variety. For each trait, the variety average, coefficient of variation (CV), statistical significance and the least significant difference (LSD_d_) are given.

Variety	Heading Date (DAS)	Maturity Date (DAS)	Plant Height at Harvest (cm)	Seed Yield per Plant (g)	TSW (g)	Seed Saponin Content (cm Foam)	No. Low-Saponin Plants	Seed Protein Content (%)	No. Plants Screened
Biobio	83 ± 0	138 ± 0	149 ± 17	65 ± 23	2.9 ± 0.2	1.9 ± 1.0	5	14.1 ± 0.6	25
Black Quinoa	66 ± 3	150 ± 8	119 ± 21	76 ± 42	3.2 ± 0.5	3.2 ± 1.3	1	13.2 ± 1.0	25
Buffy	83 ± 0	145 ± 0	138 ± 15	73 ± 24	2.7 ± 0.2	2.6 ± 0.6	1	13.6 ± 0.6	25
Chadmo	77 ± 9	169 ± 7	145 ± 37	56 ± 39	1.6 ± 0.2	1.6 ± 0.8	9	12.3 ± 1.3	22
Cherry Vanilla	83 ± 0	145 ± 0	147 ± 21	54 ± 22	2.7 ± 0.2	1.3 ± 0.9	8	14.3 ± 0.9	25
Cocoa Cherry	83 ± 0	147 ± 0	158 ± 24	90 ± 40	2.9 ± 0.3	3.7 ± 1.5	1	13.1 ± 0.6	25
Colorado BS 25	71 ± 8	174 ± 11	164 ± 31	75 ± 63	3.0 ± 0.4	2.2 ± 2.1	9	14.0 ± 1.9	22
Fingerhead GP	90 ± 0	163 ± 6	184 ± 42	49 ± 30	2.6 ± 0.5	2.7 ± 1.8	7	15.0 ± 1.7	23
French Vanilla	90 ± 0	158 ± 0	154 ± 16	80 ± 44	3.1 ± 0.3	1.9 ± 1.4	6	13.6 ± 0.5	25
Golden Afternoon	66 ± 0	147 ± 0	147 ± 18	69 ± 33	2.4 ± 0.1	1.5 ± 1.1	10	14.0 ± 1.0	25
Incred White	72 ± 8	125 ± 0	136 ± 23	61 ± 36	2.4 ± 0.4	1.1 ± 1.1	16	13.6 ± 1.3	25
Ivory	70 ± 3	138 ± 0	146 ± 12	137 ± 51	2.9 ± 0.1	2.7 ± 0.8	4	12.4 ± 0.6	22
Kaslala	67 ± 1	125 ± 0	125 ± 21	52 ± 31	2.6 ± 0.3	2.2 ± 1.7	7	13.9 ± 1.5	24
Kcoito	66 ± 0	151 ± 6	115 ± 11	62 ± 34	2.6 ± 0.2	3.0 ± 1.0	3	14.3 ± 1.1	24
Magenta sunset	68 ± 3	125 ± 0	126 ± 21	60 ± 39	2.2 ± 0.2	0.8 ± 0.4	20	12.9 ± 1.3	23
Mint Vanilla	83 ± 0	151 ± 0	133 ± 17	71 ± 18	2.6 ± 0.2	0.9 ± 1.0	20	13.4 ± 0.6	25
Oro de Valle	67 ± 4	150 ± 7	134 ± 18	93 ± 48	3.0 ± 0.2	3.9 ± 1.0	3	12.1 ± 0.7	23
Peppermint	83 ± 0	151 ± 0	139 ± 15	63 ± 36	3.0 ± 0.2	1.1 ± 0.5	9	13.3 ± 0.6	24
Pink Nugget	83 ± 0	145 ± 0	170 ± 16	47 ± 19	2.8 ± 0.3	5.7 ± 1.5	1	16.1 ± 0.9	25
Red Head	83 ± 0	151 ± 0	163 ± 19	51 ± 30	3.1 ± 0.2	1.5 ± 0.4	3	13.8 ± 0.9	23
Red Nugget	83 ± 0	151 ± 0	167 ± 13	60 ± 18	2.7 ± 0.2	3.3 ± 1.7	3	15.4 ± 0.9	22
Temuco	67 ± 0	125 ± 0	119 ± 18	42 ± 18	1.8 ± 0.2	1.1 ± 0.6	11	14.3 ± 0.8	24
White Nugget	83 ± 0	152 ± 0	165 ± 18	55 ± 22	2.8 ± 0.2	5.2 ± 1.2	2	15.6 ± 0.6	23
White Spike	83 ± 0	152 ± 0	144 ± 25	68 ± 40	2.9 ± 0.2	3.1 ± 1.6	1	14.6 ± 0.6	25
Zeno	63 ± 1	116 ± 0	90 ± 10	39 ± 15	3.6 ± 0.1	1.9 ± 0.6	2	14.8 ± 0.5	24
Variety average	76 ± 2	146 ± 3	143 ± 4	66 ± 4	2.7 ± 0.1	2.4 ± 0.3	162 (total)	13.9 ± 0.2	598 (total)
CV (%)	1.9	1.6	15	54	9.9	49		7.2	
Significance	***	***	***	***	***	***		***	
LSD_d_	20	28	47	47	3.0	1.0		2.3	

Significant differences between varieties were assessed with one-way ANOVA; ***: *p* < 0.001; DAS: days after sowing; CV: coefficient of variation; LSD_d_: least significant difference [26].

**Table 4:** Pearson correlation coefficients between important phenological and productivity traits: heading date, maturity date, plant height at seed harvest, seed yield per plant, seed saponin content, thousand-seed weight (TSW) and seed crude protein content.

	Maturity Date	Plant Height	Seed Yield	Saponin Content	TSW	Protein Content
Heading date	0.49 *	0.73 *	−0.08	0.17	−0.11	0.29
Maturity date		0.63 ***	0.18	0.23	0.02	−0.05
Plant height at seed harvest			0.08	0.37	−0.01	0.30
Seed yield per plant				0.12	0.24	−0.60 **
Saponin content					0.30	0.43 *
TSW						0.13

Statistical significance is indicated as follows: *: *p* < 0.05; **: *p* < 0.01; ***: *p* < 0.001. Correlations indicated in bold are statistically significant.

**Table 5:** Performance of twelve SSR markers organized in three multiplex PCR sets a, b and c: type of fluorescent label used for each marker and length of the SSR fragments (bp) amplified in five plants of three varieties Zeno, Black Quinoa and Oro de Valle.

Multiplex	Marker	Fluorescent Label	SSR Fragments Observed in		
SSR Set			Zeno	Black Quinoa	Oro de Valle
a	QCA071 ^F^	FAM	142	152	137
a	QAAT069	PET	195	195/200	198
a	QCA107	NED	155	155/157	155
a	QCA024	VIC	226	236/238/255	225/226
b	QATG019	FAM	163	163/166	162
b	KGA003	PET	160/161	160	160
b	QCA057	NED	184/185	168/186	187
b	QCA037	VIC	179	177/187	187
c	QAAT024 ^F^	FAM	235	195/235	222/235
c	QAAT076 ^F^	PET	181	168/169/173	169/172
c	KGA027 ^F^	NED	144	144/152	144
c	QAAT050 ^F^	VIC	197	194/197/210/212	197

^F^ These markers were chosen for the final multiplex SSR set.

**Table 6:** Fraction of progeny for which the true father could be identified through paternity analysis using our final multiplex SSR set paternity test. Success rate for our paternity test on five series of crosses between variety pairs (encoded) performed in the framework of breeding activities.

Variety Pair	No. of Seed Lots Used (♀ × ♂) *	No. Pair Crosses Performed	No. Progeny Screened	Success Rate (%)	True F_1_ Identified (%)
1	3 × 3	24	232	92	17
2	1 × 14	50	453	77	17
3	1 × 2	5	47	96	9
4	1 × 3	7	44	93	14
5	1 × 2	5	68	97	41

* Number of single-plant seed lots harvested in the variety trial described herein that was used to generate parents for the pair crosses (♀ × ♂).

**Table 7:** Quinoa material used in this study, with the variety name, the source where the seed was obtained from and the country of origin.

Variety Name	Seed Source	Origin
Biobio	Wild Garden Seeds, Philomath, OR, USA	Oregon, USA
Buffy	Wild Garden Seeds, Philomath, OR, USA	Oregon, USA
Cherry Vanilla	Wild Garden Seeds, Philomath, OR, USA	Oregon, USA
Cocoa Cherry	Wild Garden Seeds, Philomath, OR, USA	Oregon, USA
Fingerhead GP	Wild Garden Seeds, Philomath, OR, USA	Oregon, USA
French Vanilla	Wild Garden Seeds, Philomath, OR, USA	Oregon, USA
Golden afternoon	Wild Garden Seeds, Philomath, OR, USA	Oregon, USA
Incred White	Wild Garden Seeds, Philomath, OR, USA	Oregon, USA
Ivory	Wild Garden Seeds, Philomath, OR, USA	Oregon, USA
Kaslala	Wild Garden Seeds, Philomath, OR, USA	Oregon, USA
Mint Vanilla	Wild Garden Seeds, Philomath, OR, USA	Oregon, USA
Peppermint	Wild Garden Seeds, Philomath, OR, USA	Oregon, USA
Pink Nugget	Wild Garden Seeds, Philomath, OR, USA	Oregon, USA
Red Head	Wild Garden Seeds, Philomath, OR, USA	Oregon, USA
Red Nugget	Wild Garden Seeds, Philomath, OR, USA	Oregon, USA
White Nugget	Wild Garden Seeds, Philomath, OR, USA	Oregon, USA
White Spike	Wild Garden Seeds, Philomath, OR, USA	Oregon, USA
Black Quinoa	De Nieuwe tuin, De Klinge, Belgium	Colorado, USA
Kcoito	De Nieuwe tuin, De Klinge, Belgium	Bolivia
Oro de Valle	De Nieuwe tuin, De Klinge, Belgium	Oregon, USA
Chadmo	Association Kokopelli, Le Mas d’Azil, France	Chile
Colorado Black Shelly 25	Association Kokopelli, Le Mas d’Azil, France	Colorado, USA
Magenta sunset	Association Kokopelli, Le Mas d’Azil, France	Oregon, USA
Temuco	Association Kokopelli, Le Mas d’Azil, France	Chile
Zeno	Hohenheim University, Stuttgart, Germany	Austria

**Table 8:** Overview of the phenotypic traits considered in this study, with their respective observation dates, units and scores.

Trait	Units	Observation Date (DAS)	Score 1	Score 2	Score 3	Score 4	Score 5	Score 6	Score 7	Score 8	Score 9	Score 10
Foliage color ^S^	score	62	light green	medium green	dark green	red	purple					
Foliage glaucosity ^S^	score	70	absent	weak	medium		strong					
Stem color ^S^	score	69	white	green	yellow	purple	NA					
Presence of stem stripes ^S^	score	69	absent								present	
Pigmentation of leaf axil ^S^	score	69	absent	very weak	weak		medium		strong			
Leaf size ^S^	score	76			small		medium		large			
Leaf dentation ^S^	score	68	absent	weak	medium		strong					
Growth habit ^S^	score	83	not branched		sparse		substantial		no main panicle			
Inflorescence color ^S^	score	76–90	white	green	yellow	orange	pink	purple				
Panicle shape ^S^	score	83–90	glomerulate		intermediate		amarantiform					
Panicle width ^S^	score	84			narrow		medium		broad			
Panicle density ^S^	score	83–104	loose		intermediate		axis rarely visible		compact			
Panicle color ^S^	color	83–90	white	purple	red	pink	yellow	orange	brown	gray	black	green
Seed color ^S^	color		white	beige	yellow	brown	red	black				
Heading date ^S^	DAS	60–74										
Maturity date ^S^	DAS	116–185										
Plant height at harvest ^S^	cm	109										
Seed weight	g											
TSW	g											
Seed saponin content ^K^	cm foam											
No. of low-saponin plants	#											
Seed crude protein content	%											

^S^: Full description for these phenotypic traits is given in Stanschewski et al. [25]. ^K^: Full description of the saponin foam test is given in Koziol et al. [10]. TSW: thousand-seed weight; DAS: days after sowing.

**Table 9:** Details of the SSR markers screened in this study, with their respective primers, amplification ranges, linkage groups [36,38], number of alleles, and the fluorescent label used by Christensen et al. [35].

Marker	Forward Primer	Reverse Primer	Amplification Range	Linkage Group	Number of Alleles	Fluorescent Label Used
**KGA03 ^b^**	attgccgacaatgaacgaat	atgtaaatggcatgtcccaac	140–182	26	5 [36], 21 [35]	HEX *
**KGA27 ^c^**	ttgtacagaggaagtggcaaga	catcttacagctctggctttcc	126–158	NA	6 [36], 16 [35]	NED
**QAAT024 ^c^**	accataacagcacccacctt	agggatcaatcttgttcattca	201–257	15	6 [37], 20 [35]	FAM
**QAAT050 ^c^**	ggcacgtgctgctactcata	tatggcgaatggttaatttgc	158–246	13	9 [37], 27 [35]	VIC
**QAAT069 ^a^**	gtttcctttgaggcttggac	ggatttgtacgaatagttgggatt	193–266	NA	4 [37], 15 [35]	NED
QAAT070	tgaacaggatcgtcatagtcaa	cgttcatcatctgacccaat	158–208	NA	7 [37], 17 [35]	NED
QAAT074	atggaacacccatccgataa	atgcctatcctcatcctcca	169–224	NA	9 [37], 16 [35]	FAM
**QAAT076 ^c^**	gcttcatgtgttataaaatgccaat	tctcggcttcccactaatttt	145–227	NA	8 [37], 27 [35]	HEX *
QAAT078	agcgaaggaaatttggaact	taacgatacgctccaaggaa	183–226	12	5 [37], 13 [35]	HEX *
**QATG019 ^b^**	ccaaacaaagacaataaggaaacc	cgaggttgaaggagattcca	175–193	11	6 [37], 7 [35]	FAM
QCA019	tttcatcactcgaccgtatagc	agggtgactgttacacccaaa	183–212	2	3 [37], 6 [35]	VIC
**QCA024 ^a^**	agatgagcttgaatcattacatc	tacatactgtaaatcatgccaaa	235–254	NA	3 [37], 7 [35]	VIC
**QCA037 ^b^**	ccgttcttccagaccaattc	tcatgagccacttcatacacg	186–206	18	5 [37], 9 [35]	VIC
QCA038	catttcccaaactgcatgaat	atgtgtgttgcgtgtgagtg	198–209	NA	4 [37], 5 [35]	VIC
QCA048	acaatacatacataacccaatattcaa	tggaaatgtcactatgattgga	246–258	12	3 [37], 6 [35]	FAM
**QCA057 ^b^**	tgcaaggaaaccatctttgg	tgcctcacagtcacacctaca	196-240	2	4 [37], 8 [35]	NED
**QCA071 ^a^**	aacaacgaaattacgagaatgtca	tctcacgagagtcttccccta	140–177	NA	4 [37], 16 [35]	FAM
**QCA107 ^a^**	acaggctgtgggtccactt	tcaagcaatactcaccttgtgg	153–167	1	5 [37], 5 [35]	NED

Markers in bold were tested in multiplex PCR sets a, b and c, respectively. Number of alleles according to the literature [35,36,37]. *: HEX fluorescent labels used in the literature were replaced by PET labels in this present work.

## Data Availability

All available data are included in the published paper.

## Abbreviations

DAS: days after sowing; NIRS: near-infrared reflectance spectroscopy; PCR: polymerase chain reaction; SSR: simple sequence repeat; TSW: thousand-seed weight.
